# Geranylgeranylacetone attenuates fibrogenic activity and induces apoptosis in cultured human hepatic stellate cells and reduces liver fibrosis in carbon tetrachloride-treated mice

**DOI:** 10.1186/s12876-018-0761-7

**Published:** 2018-02-27

**Authors:** Takemasa Senoo, Ryu Sasaki, Yuko Akazawa, Tatsuki Ichikawa, Satoshi Miuma, Hisamitsu Miyaaki, Naota Taura, Kazuhiko Nakao

**Affiliations:** 1 Department of Gastroenterology and Hepatology, Nagasaki University Graduate School of Biomedical Sciences, 1-7-1 Sakamoto, Nagasaki, 852-8501 Japan; 2 Department of Clinical Oncology Unit of Translational Medicine, Nagasaki University Graduate School of Biomedical Sciences, 1-7-1 Sakamoto, Nagasaki, 852-8501 Japan; 3 Department of Gastroenterology, Nagasaki Harbor Medical Center, 6-39 Shinchi, Nagasaki, 850-8555 Japan; 4 Department of Gastroenterology and Hepatology, Nagasaki University Hospital, 1-7-1 Sakamoto, Nagasaki, 852-8501 Japan

**Keywords:** Geranylgeranylacetone, Hepatic stellate cells, Liver fibrosis, Apoptosis

## Abstract

**Background:**

Geranylgeranylacetone (GGA), an anti-ulcer drug widely used in Japan, has attracted interest because of its various therapeutic effects. Therefore, we investigated the effects of GGA on human hepatic stellate cells (HSCs) in vitro and in a mouse model of liver fibrosis.

**Methods:**

LX2, an immortalized human HSC line, was cultured and treated with GGA at concentrations up to 0.5 mM. After GGA treatment, changes in cellular morphology, apoptosis, and fibrosis-related gene expression were assessed. Male C57BL/6 J mouse model of carbon tetrachloride (CCl_4_)-induced liver fibrosis was treated with GGA. Liver fibrosis was evaluated using Sirius red staining and immunohistochemistry for α-smooth muscle actin (SMA).

**Results:**

GGA decreased the density of LX2 and primary human hepatic stellate cells but not that of HepG2 cells (a human hepatoma cell line), which was employed as control. In addition, GGA decreased the expression of fibrogenic genes and increased that of C/EBP homologous protein (CHOP). It also induced endoplasmic reticulum (ER) stress and increased apoptosis. CHOP knockdown, however, failed to suppress the GGA-induced decrease in LX2 cell density, suggesting the involvement of additional molecules in ER stress–associated apoptosis. Expression of death receptor 5, mitogen-activated protein kinase, heat shock protein 70, and Akt, all of which affect the activity of stellate cells, was unchanged in relation to LX2 cell fibrogenic activity. In the mouse model of liver fibrosis, GGA decreased the extent of Sirius red staining and SMA expression.

**Conclusions:**

GGA attenuated fibrogenic activity and induced apoptosis in cultured human HSCs, and suppressed liver fibrosis in mice, suggesting its potential as an agent for treating liver fibrosis.

**Electronic supplementary material:**

The online version of this article (10.1186/s12876-018-0761-7) contains supplementary material, which is available to authorized users.

## Background

Hepatic fibrosis can be caused by various factors, including viral infection, alcohol abuse, drug toxicity, hereditary metabolic disorders, and autoimmune diseases. Regardless of its etiology, hepatic fibrosis ultimately leads to liver cirrhosis and hepatoma development. It is widely recognized that hepatic stellate cells (HSCs) play an important role in hepatic fibrogenesis. To promote hepatic fibrosis, HSCs must undergo an activation process followed by the overexpression of fibrogenic genes, including collagen or α-smooth muscle actin (SMA), as well as a phenotypic change from an oval to a spindle shape [1]. Therefore, inhibiting HSC activation is essential for the effective treatment of hepatic fibrosis.

Several studies have shown that suppressing HSC activation attenuates hepatic fibrosis [2–4]. The underlying mechanisms for the suppression of HSC inactivation or death include inhibition of the renin-angiotensin system, suppression of the phosphatidylinositol 3-kinase (PI3K)–Akt pathway, activation of mitogen-activated protein kinase (MAPK), upregulation of death receptor 5 (DR5), and apoptosis associated with endoplasmic reticulum (ER) stress [5–9]. However, the role of these pathways in HSCs remains controversial. For example, ER stress has been reported to induce fibrogenic activity in HSCs [10], but other studies found that HSC death occurred through ER stress–mediated apoptosis [11, 12]. These findings suggest that HSC fate may depend on the magnitude and type of activated stress in the ER. To elucidate the mechanisms of hepatic fibrosis with the goal of developing new therapeutic options, further research on the identification of effective and safe antifibrogenic agents is crucial.

Geranylgeranylacetone (GGA) is an anti-ulcer drug that has been used for many years in Japan. It has recently attracted additional interest for its various effects in addition to its original virtues. For example, several studies have demonstrated that GGA has the ability to induce the expression of heat shock protein (HSP) families in various organs, including the liver [13–15]. In vivo, He et al. showed that GGA suppressed extracellular matrix (ECM) protein deposition in rat liver specimens and regulated the progression of hepatic fibrosis through the upregulation of HSP70 expression [16]. Another study showed that GGA induced ER stress in rat mesangial cells [17]. However, the molecular mechanisms of these beneficial effects on HSCs are largely unknown. In this study, we evaluated whether GGA can directly attenuate fibrogenic activity in cultured human HSCs and reduce hepatic fibrosis in animals other than rats.

## Methods

### Cell culture

The human immortalized HSC line LX2, generously donated by Dr. Scott L. Friedman (Mount Sinai School of Medicine, NY, USA), was cultured with high-glucose Dulbecco’s Modified Eagle Medium (DMEM) containing 10% fetal bovine serum and 1% penicillin-streptomycin. The human hepatoma cell line HepG2 (American Type Culture Collection, Manassas, VA, USA) was cultured with high-glucose DMEM containing 10% fetal bovine serum and 1% penicillin-streptomycin. The primary human hepatic stellate cells (HHSteC, ScienCell Research Laboratories, CA, USA) isolated from the human liver were cultured in stellate cell medium (ScienCell Research Laboratories) supplemented with 2% fetal bovine serum and 1% penicillin-streptomycin. All cell cultures were maintained at 37 °C in a humidified atmosphere containing 5% CO_2_.

### Quantification of apoptosis

Cells were plated in a 4-well chamber slide (Thermo Fisher Scientific Inc., Boston, MA, USA) and incubated overnight. The following day, fresh medium was added with various reagents (described in the Results section). Twenty-four hours later, cells were washed with cold phosphate-buffered saline (PBS) and fixed in 4% paraformaldehyde for 15 min at 37 °C, followed by staining with 4′,6-diamidino-2-pheny-indole (DAPI). The percentage of apoptotic cells was determined by counting the number of DAPI-stained condensed nuclei and the total number of nuclei per field (percentages were the mean of three randomly chosen fields per condition).

### RNA extraction and real-time PCR

LX2 cells were incubated in 100 mm plates for 24 h under different experimental conditions, and total RNA was extracted with a commercially available kit (GenElute™ Mammalian Total RNA Miniprep Kit, Sigma-Aldrich, St. Louis, MO, USA) according to the manufacturer’s instructions. Extracted RNA was measured using NanoDrop 1000 (Nanodrop Technologies, Wilmington, DE, USA), and 2 μg of total RNA was reverse transcribed into cDNA using a reverse transcription kit (Thermo Fisher Scientific Inc.). cDNA was amplified by PCR with the following gene-specific primers: SMA (forward) 5’-CTGTTCCAGCCATCCTTCAT-3′ (reverse) 5’-CCGTGATCTCCTTCTGCATT-3′, type 1 collagen (forward) 5’-CCTCAAGGGCTCCAACGAG-3′ (reverse) 5’-TCAATCACTGTCTTGCCCCA-3′, C/EBP homologous protein (CHOP) (forward) 5’-GCGCATGAAGGAGAAAGAAC-3′ (reverse) 5’-TCACCATTCGGTCAATCAGA-3′, activating transcription factor 6 (ATF6) (forward) 5’-AGCATGTTCCTGAGGAGTTGG-3′ (reverse) 5’-AGGCTTATCTTCCTTCAGTGGC-3′, Bip (forward) 5’-CGTGTTCAAGAACGGCCG-3′ (reverse) 5’-CGTAGACAGTACGACAGCAACTGT-3′, glyceraldehyde-3-phosphate dehydrogenase (GAPDH) (forward) 5’-CATGGGTGGAATCATATTGGAA-3′ (reverse) 5’-GAAGGTGAAGGTCGGAGT-3′.

### Western blotting analysis

After treatment with various conditions (described in the Results section), cultured cells were washed with cold PBS and lysed on ice using radioimmunoprecipitation assay (RIPA) buffer. Cell lysates were centrifuged for 15 min at 14000 rpm at 4 °C, and whole cell protein was extracted. Extracted proteins were normalized using a bicinchoninic acid (BCA) assay kit (Thermo Fisher Scientific Inc.). Total protein samples were separated using sodium dodecyl sulfate polyacrylamide gel electrophoresis (SDS-PAGE) and transferred electrophoretically to nitrocellulose membranes (Bio-Rad, Hercules, CA, USA). After blocking with 5% milk, membranes were incubated with primary antibodies against SMA (Abcam, Cambridge, UK), β-actin, CHOP, Bip, ATF6, phosphorylated extracellular signal regulated kinase (phospho-ERK), eukaryotic initiation factor (eIF)2α, phospho-eIF2α, c-Jun N-terminal kinase (JNK), phospho-JNK, p38 mitogen-activated protein kinase (MAPK), phospho-p38MAPK, poly(ADP-ribose) polymerase (PARP), cleaved-PARP, Akt, phospho-Akt, HSP70 (Cell Signaling technology, Beverly, MA, USA) and type 1 collagen (Santa Cruz Biotechnology, Dallas, TX, USA). Membranes were then incubated with horseradish peroxidase-conjugated secondary antibody. Protein bands were detected by chemiluminescence.

### Chemicals and reagents

GGA was purchased from Wako Co. (Osaka, Japan) and prepared in pure ethanol. The proteasome inhibitor MG132 was purchased from Merck Millipore (Darmstadt, Germany) and used at a concentration of 10 μM in all experiments.

### Immunofluorescent microscopy

SMA expression in LX2 cells was evaluated by immunofluorescent microscopy. Cells were plated in 4-well chamber slides (Thermo Fisher Scientific Inc.) and treated with GGA. Cells were then washed with cold PBS and fixed with 4% paraformaldehyde for 15 min at 37 °C. Next, the cells were washed with PBS and permeabilized by 0.0125% CHAPS in PBS at 37 °C for 10 min. After incubation with 5% goat serum for 30 min, cells were stained with anti-SMA (Abcam) overnight at 4 °C and then incubated with Alexa Fluor 488-conjugated goat anti-rabbit IgG (Molecular Probes, Eugene, OR, USA) for 1 h at 37 °C. Immunostained slides were assessed by confocal microscopy (excitation and emission wavelengths were 488 and 507 nm, respectively).

### Small interfering (si) RNA transfection

To examine the influence of CHOP expression in LX2 cells treated with GGA, we performed experiments using siRNA transfection. RNAi was obtained from Dharmacon (Lafayette, CO, USA, CHOP siGENOME SMART pool, catalog #M-004819-03-0005) and transfected to LX2 cells according to the manufacturer’s protocol. Cells were used for experiments 24 h after transfection. To confirm knockdown of CHOP, RT-PCR was performed as described above.

### Animal models

Male C57BL/6 J mice were purchased from the Kyudo Company (Saga, Japan). A total of 12 male mice were housed under standard animal laboratory conditions, with controlled temperature (22 ± 1 °C), humidity (65 ± 5%), and 12 h light/dark cycles with free access to food and water, in a specific-pathogen-free-grade animal room. Mice were used for experimentation at 6 weeks of age. The animal experimentation protocols were approved by the University of Nagasaki Animal Studies Committee. This study also followed the guidelines of the National Institutes of Health Guide for the Care and Use of Laboratory Animals. The mice were randomly divided into two groups: the carbon tetrachloride (CCl_4_) group and the CCl_4_ + GGA group. There was no difference in the mean body weight between the two groups. In the CCl_4_ group, liver fibrosis was induced in the mice by oral administration of 400 ml/l CCl_4_ salad oil solution, with a single dose of 0.5 μl/g/body weight twice per week (*n* = 6). In the CCl_4_ + GGA group, mice received the same dose of CCl_4_ as the control group mice, along with a daily oral dose of an emulsion containing 0.4 mg/g/body weight GGA starting 4 weeks after CCl_4_ administration (n = 6). After 6 weeks, all mice were sacrificed by injection with pentobarbital (75 mg/kg i.p.; Kyoei Pharmaceutical Co., Chiba, Japan) following blood extraction via cardiac puncture and liver removal. The liver pieces were quickly removed and fixed in 10% neutral buffered formalin, processed routinely, and embedded in paraffin wax.

### Histopathology

The mouse liver sections were stained with Sirius red and immunohistochemical analysis was performed for SMA. Sirius red staining was performed using “Picrosirius Red Stain Kit” (catalog #24901–250, Polysciences, Inc., Warrington, PA, USA). SMA immunostaining was performed with rabbit an anti-actin SMA polyclonal antibody (catalog #bs-0189R, Bioss Antibodies Inc., Woburn, MA, USA). ImageJ software (version 1.48, National Institutes of Health, NIH, USA) was used to analyze the stained area percentage.

### Statistical analysis

Quantitative data are expressed as means ± standard deviation (SD) of at least three independent experiments. We used the Student’s *t*-test to compare the two values. Differences between groups were analyzed by analysis of variance (ANOVA) with a post-hoc Dunnett’s multiple comparison tested. *P*-values less than 0.05 were considered to be statistically significant.

## Results

### GGA decreased density of HSCs

**Fig. 1:**
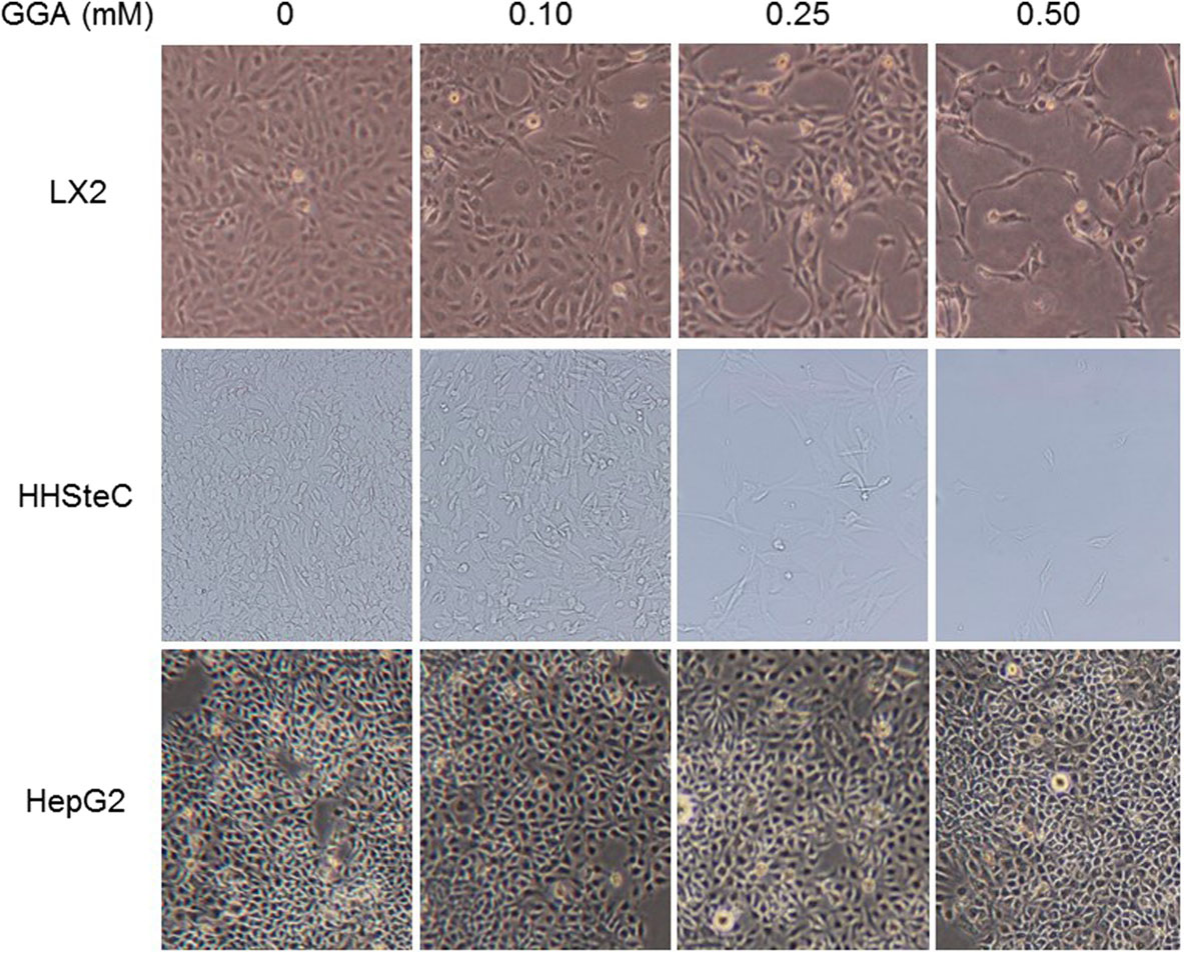
Geranylgeranylacetone (GGA) decreased LX2 and HHSteC density in a concentration-dependent manner. After treatment with GGA (0.10–0.50 mM) for 24 h, three different cell lines, LX2 cells, HHSteCs and HepG2 cells, were observed by phase-contrast microscopy. There were no significant morphological changes or cell density decrease in HepG2 cells

We monitored LX2 cells treated with GGA using phase-contrast microscopy and observed a concentration-dependent decrease in cell density (Fig. 1). Similarly, GGA reduced the density of HHSteCs in a concentration-dependent manner. In contrast, HepG2 cells showed no significant morphological or density changes with GGA treatment.

### GGA suppressed fibrosis-related gene and protein expression in LX2 cells

**Fig. 2:**
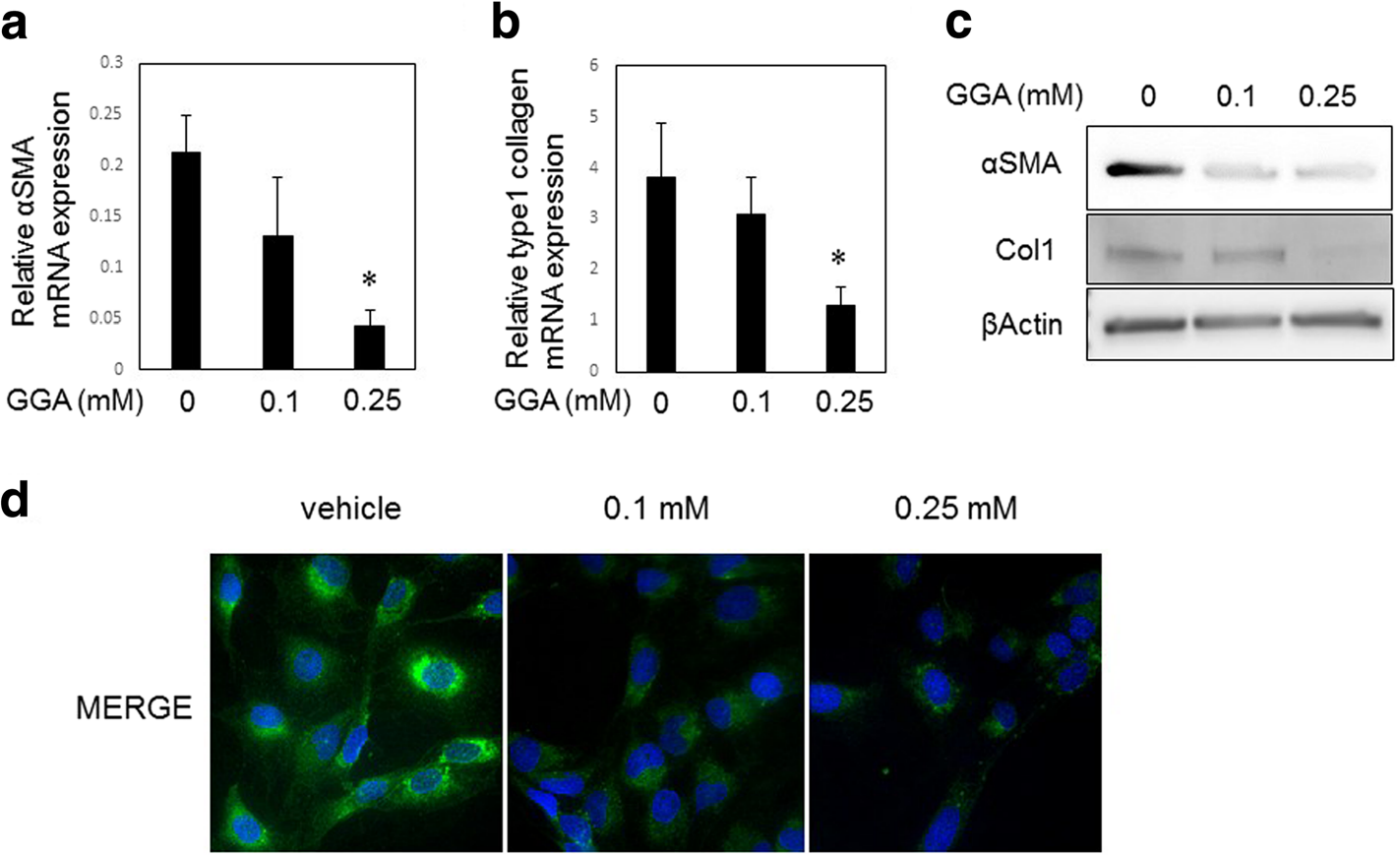
GGA suppressed activation and fibrogenesis of LX2 cells in a dose-dependent manner. LX2 cells were incubated in the presence or absence of GGA (0.10–0.50 mM) for 24 h, and the expression of the fibrosis-related proteins, α-smooth muscle actin (SMA) and type 1 collagen was assessed by real-time polymerase chain reaction (PCR) (**a**, **b**) and western blotting (**c**). GGA decreased SMA expression in LX2 cells as measured using immunofluorescent microscopy. GGA treatment was performed for 24 h at the indicated concentrations. **d** The number of all experiments was *n* = 3. *P < 0.05

To evaluate the effects of GGA on the expression of the fibrosis markers SMA and type 1 collagen, we performed real-time PCR, western blotting analysis, and immunofluorescent microscopy using LX2 cells. Cells were treated with GGA (0, 0.1, and 0.25 mM) for 24 h. GGA treatment decreased SMA and type 1 collagen mRNA expression in a concentration-dependent manner and significantly suppressed these fibrosis markers at 0.25 mM (Fig. 2a, b). Western blotting analysis showed that the expression of the fibrosis-related proteins decreased in LX2 cells treated with GGA (Fig. 2c). Immunofluorescence analysis of LX2 cells treated with GGA confirmed the previously observed SMA expression suppression (Fig. 2d).

### GGA induced apoptosis of HSCs

**Fig. 3:**
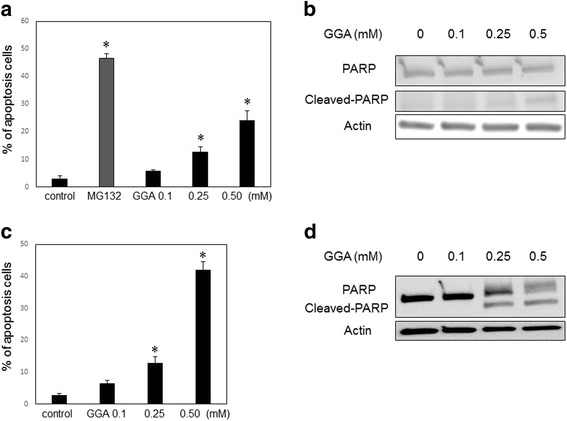
GGA induced apoptosis in LX2 and HHSteCs. After 24 h treatment with GGA (0.10–0.50 mM) or the positive control MG132 (10 μM), the percentage of apoptotic cells was counted using DAPI staining (**a**), and western blotting was performed for the apoptosis marker poly(ADP-ribose) polymerase (PARP) and cleaved-PARP (**b**) in LX2 cells. Similarly, after 24 h treatment with GGA (0.10–0.50 mM), the percentage of apoptotic HHSteCs was counted using DAPI staining (**c**), and western blotting was performed for PARP and cleaved-PARP (**d**) in HHSteCs. The number of all experiments was n = 3. *P < 0.05

To investigate if cell death is involved in the regression of stellate cell density induced by GGA, we evaluated apoptosis in cells subjected to various GGA treatment conditions using DAPI staining. DAPI staining revealed chromatin condensation in nuclei and fragmentation in a concentration-dependent manner (Fig. 3a). In addition, PARP cleavage was observed by immunoblotting in LX2 cells treated with GGA (Fig. 3b). Taken together, these data suggest that GGA not only reduces fibrotic action within LX-2 cells, but also induces apoptosis. In addition, we also observed the apoptosis of HHSteCs treated with GGA using DAPI staining and western blotting for PARP. (Fig. 3c and d).

### GGA induced endoplasmic reticulum (ER) stress in LX2 cells

**Fig. 4:**
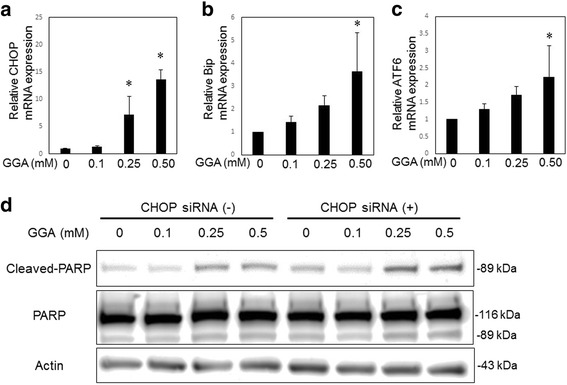
GGA induced endoplasmic reticulum stress in LX2 cells. C/EBP homologous protein (CHOP), Bip, and activating transcription factor 6 (ATF6) mRNA expression were assessed by real-time PCR in LX2 cells treated with GGA (**a**, **b**, **c**). The difference in PARP cleavage between control LX2 cells and LX2 cells transfected with CHOP siRNA was assessed by western blotting analysis (**d**). In all experiments, LX2 cells were incubated in the presence or absence of GGA (0.10–0.50 mM) for 24 h. All experiments had n = 3. *P < 0.05

To further investigate the effect of GGA on HSCs, we determined if ER stress plays a role in GGA-induced apoptosis. First, we performed RT-PCR in LX2 cells treated with GGA, as shown in Fig. 4a-c. GGA significantly increased the mRNA expression levels of the ER stress markers ATF6, Bip, and CHOP in a concentration-dependent manner. Notably, the relative expression of CHOP, which plays a central role in ER-stress mediated apoptosis, increased more than 10 times after treatment with 0.5 mM GGA (Fig. 4a). These data demonstrate that GGA induced ER stress in LX2 cells.

### Knockdown of CHOP failed to suppress GGA-induced decrease of LX2 cells

To elucidate whether GGA-induced apoptosis was associated with ER stress, we transfected CHOP siRNA into LX2 cells. Transfection was performed as described above, and we confirmed that the expression of CHOP mRNA was suppressed in siRNA-transfected LX2 cells. Next, to detect apoptosis induction, we observed PARP cleavage by western blot. PARP cleavage was not significantly different between CHOP siRNA-transfected LX2 cells and control cells (Fig. 4d).

### Effect of GGA on LX2 was not associated with MAPK expression

Members of the MAPK, JNK, and p38-MAPK pathways are known to be associated with HSC activation and liver fibrosis progression. Kluwe et al. reported that JNK phosphorylation was upregulated in a rat model of liver cirrhosis and that inhibiting JNK phosphorylation resulted in HSC inactivation [18]. Another study found that p38-MAPK induced HSC activation via SMAD3 phosphorylation [19]. To elucidate the mechanism of GGA-induced LX2 cell apoptosis, we measured phosphorylated JNK and p38-MAPK protein levels. We found that the phosphorylation of these proteins was not significantly changed in LX2 cells after 24-h GGA treatment (Additional file 1: SA).

### GGA suppressed DR5 mRNA expression in LX2 cells

Previously, it was shown that DR5 overexpression induced apoptosis in LX2 cells [20]. Thus, we measured the expression of DR5 in LX2 cells treated with GGA using RT-PCR. GGA treatment suppressed rather than exacerbated DR5 levels. After treatment with GGA for 24 h, DR5 mRNA expression was significantly suppressed in a concentration-dependent manner in LX2 cells (Additional file 1: SB). Taimr et al. reported that DR5 mRNA expression increased during spontaneous activation in HSC [20]. Thus, we speculated that the suppression of DR5 expression after GGA treatment may be the result of LX2 cell inactivation.

### GGA-induced decreases in LX2 cell density were independent of the PI3K/Akt pathway

Yu et al. showed using LX2 cells that the PI3K/Akt signaling pathway is important in the process of liver fibrosis [21]. Therefore, we examined changes in the PI3K/Akt signaling pathway in LX2 cells treated with GGA. Akt phosphorylation in LX2 cells after GGA treatment for 24 h was measured by western blot. We found that GGA treatment enhanced rather than suppressed Akt phosphorylation (Additional file 1: SC), suggesting that GGA-induced decreases in LX2 cell density were independent of the PI3K/Akt pathway.

### GGA did not induce HSP70 upregulation in LX2 cells

GGA treatment is known to induce upregulation of HSP family proteins in several organs. Previously, it was shown that GGA treatment in mice upregulated HSP70 levels in a liver specimen and was associated with suppression of liver fibrosis [16]. Therefore, we measured HSP70 expression in LX2 cells treated with GGA by western blot, and found that treatment with GGA for 24 h did not significantly change HSP70 expression compared to controls (Additional file 1: SD).

### GGA suppressed liver fibrosis in the mouse model

**Fig. 5:**
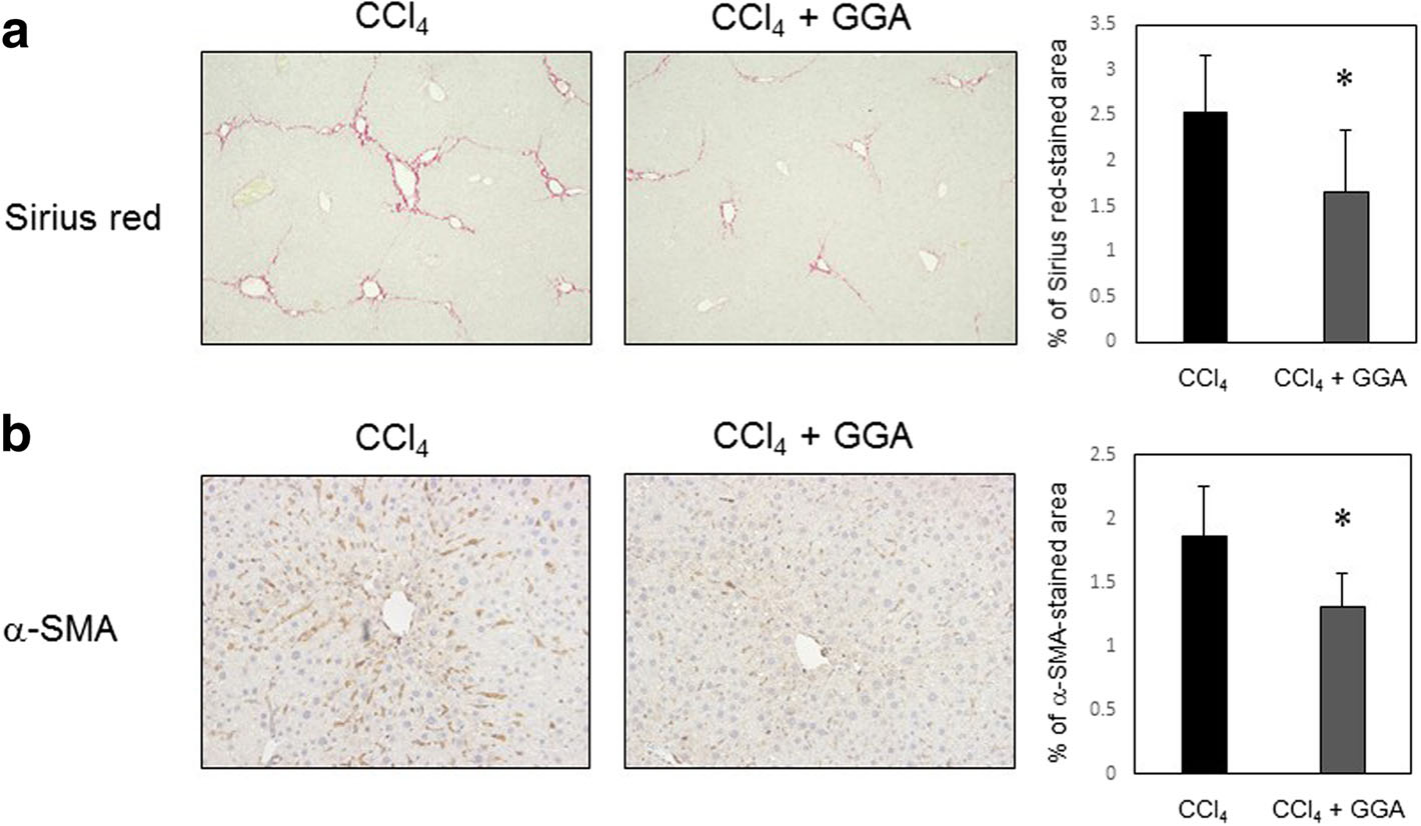
Histopathological analysis of liver specimen of male C57BL/6 J mice treated with CCl_4_ or CCl_4_ and GGA (*n* = 6 each). Sirius Red staining of liver tissue sections (200 × objective) and morphometric analysis of Sirius Red-stained area size (**a**). Immunohistochemistry for α-smooth muscle actin (SMA) in liver tissue sections (200 × objective) and morphometric analysis of SMA-positive area size (**b**). Bar graphs represent means ± SEM. * = P < 0.05

Liver fibrosis development was investigated in CCl_4_-treated mice through pathological examinations. Notable liver fibrosis, marked by positive Sirius Red staining, was observed in CCl_4_-treated mice (Fig. 5a). However, fibrosis was significantly suppressed in the CCl_4_ + GGA group compared to that in the CCl_4_ group (Fig. 5a). Further, in agreement with Sirius Red staining data, the SMA-positive area percentage was significantly lower in the CCl_4_ + GGA group livers (Fig. 5b). These data suggested that GGA reduced the fibrogenic activity of HSCs in vivo*.*

## Discussion

In the present study, we demonstrated that directly treatment of cultured human HSCs with GGA could attenuate fibrogenic activity and induce apoptosis. We used LX2 cells as our human HSC model because LX2 cells retain key features of primary HSCs and show viability in a serum-free media with high transfectability [22]. The density of the LX2 and HHSteCs was significantly decreased after treatment with GGA, as shown in Fig. 1, a change that was not observed in HepG2 cells. The results may indicate that the effect of GGA varies depending on cell line. In terms of liver fibrosis progression, HSC activation involves switching from oval shaped, lipid storing quiescent cells to proliferative, fibrogenic contractile myofibroblasts [1, 23]. She et al. reported that the regulation of adipogenic transcriptional factors in activated HSCs caused their phenotypic reversal to quiescent HSCs [24]. In addition to phenotypic changes, alterations in the expression of fibrosis markers, such as SMA and collagen 1, can also reflect HSC activation. In this study, we found that GGA treatment also suppressed fibrosis marker expression in LX2 cells in a concentration-dependent manner (Fig. 2). In addition, GGA treatment increased LX2 cell apoptosis similarly in the HHSteCs (Fig. 3). Based on these findings, we concluded that apoptosis was the main cause of the GGA-induced decrease in cell density of HSCs. To elucidate the mechanism of GGA-mediated apoptosis, we evaluated several pathways known to be associated with HSC apoptosis or activation. Since MAPK activation had been reported to play a role in stellate cell apoptosis [18], we first tested whether activation of JNK and p38 MAPK was involved in GGA-mediated apoptosis. Interestingly, MAPK activation was not induced in LX2 cells treated with GGA in our study. Likewise, we did not observe an upregulation of DR5 expression or any change in Akt expression. Therefore, the effect of GGA on human HSCs is likely not associated with the MAPK, DR5, or Akt/PI3K pathways. The ability of GGA to induce HSP upregulation in several organs is well known [25, 26]. Although He et al. had indicated that the upregulation of HSP70 by GGA was associated with the suppression of fibrogenesis in the mouse liver [16], we did not find evidence of GGA-induced HSP70 upregulation in LX2 cells. These discrepancies may have resulted from the difference in the cell lines. A previous in vitro study showed that GGA upregulated HSP70 and inhibited the apoptosis of rat hepatocytes exposed to hydrogen peroxide [27]. GGA induced HSP70 upregulation may occur in hepatocytes, which constitute the majority of liver cells, in vivo. To our knowledge, the present study is the first to directly treat human HSCs with GGA. Our study suggested that the effect of GGA on human HSCs may not be associated with HSP expression modulation.

In the present study, GGA treatment induced ER stress in a concentration-dependent manner (Fig. 4). Interestingly, the upregulation of the expression of CHOP, a factor well known to promote apoptosis, showed marked increased. Thus, we suppressed CHOP expression using siRNA in LX2 cells treated with GGA. However, knockdown of CHOP expression did not suppress apoptosis in LX2 cells treated with GGA (Fig. 4d). It remains unclear whether the induction of ER stress has some effect on LX2 cells, and our results showed that CHOP by itself could not induce cell death, and that additional mediators may be required to successfully induce HSC death. Previous studies using HSCs or hepatocytes with the activation of death receptors suggest that JNK correlates with ER stress [20, 28]. However, we did not detect the involvement of these proteins in our study. Further investigation is required to elucidate the mechanism of the GGA-mediated apoptosis of HSCs.

A mouse model of CCl_4_-induced hepatic fibrosis was used in our study. GGA attenuated the development of CCl_4_-induced liver fibrosis in mice as shown by histochemical analysis. Our results are consistent with those of a previous report by He et al., which showed decreased fibrosis in GGA-treated rat [16]. Thus, the results, in vitro and in vivo, in the present study indicate that GGA has a potential to exerts an anti-fibrogenic effect on the liver of various animals including humans.

## Conclusions

Collectively, the results of the present study suggest that GGA could induce apoptosis in HSCs in multifactorial mechanisms and facilitate the attenuation of fibrogenic activity without severe adverse effects in vivo. GGA could potentially be beneficial for the treatment of liver fibrosis in humans.

## Additional file

Additional file 1: Regarding the activation of Janus N-terminal kinase (JNK) and p38-mitogen activated protein kinase (MAPK), no significant changes were observed in LX2 cells treated with GGA using western blotting analysis (A). GGA suppressed death receptor 5 (DR5) expression in LX2 cells. The expression of DR5 was assessed by real-time PCR. (B) The upregulation of phosphorylated Akt in LX2 cells treated with GGA was observed using western blotting analysis (C). There was no significant change in the expression of HSP70 in LX2 cells treated with GGA and control cells using western blotting analysis (D). In all experiments, LX2 cells were incubated in the presence or absence of GGA (0.10–0.50 mM) for 24 h. * = *P* < 0.05. (JPEG 73 kb)

## Abbreviations

ATF6: Activating transcription factor 6
CCl_4_: Carbon tetrachloride
CHOP: C/EBP homologous protein
DAPI: 4′6-diamino-2-pheny-indole
DMEM: Dulbecco’s Modified Eagle Medium
DR5: Death receptor 5
ECM: Extracellular matrix
eIF: Eukaryotic initiation factor
ER: Endoplasmic reticulum
ERK: Extracellular signal regulated kinase
GAPDH: Glyceraldehyde-3-phosphate dehydrogenase
GGA: Geranylgeranylacetone
HSCs: Hepatic stellate cells
HSP: Heat shock protein
JNK: c-Jun N-terminal kinase
MAPK: Mitogen-activated protein kinase
PARP: Poly(ADP-ribose) polymerase
PBS: Phosphate-buffered saline
PI3K: Phosphatidylinositol 3-kinase
SD: Standard deviation
siRNA: Small interfering RNA
SMA: α-smooth muscle actin

## References

[1] Friedman SL (2000). Molecular regulation of hepatic fibrosis, an integrated cellular response to tissue injury. J Biol Chem.

[2] Bohanon FJ, Wang X, Ding C, Ding Y, Radhakrishnan GL, Rastellini C (2014). Oridonin inhibits hepatic stellate cell proliferation and fibrogenesis. J Surg Res.

[3] Ogawa T, Kawada N, Ikeda K (2009). Effect of natural interferon alpha on proliferation and apoptosis of hepatic stellate cells. Hepatol Int.

[4] Ghazwani M, Zhang Y, Gao X, Fan J, Li J, Li S (2014). Anti-fibrotic effect of thymoquinone on hepatic stellate cells. Phytomedicine.

[5] Wang J, Xu F, Zhu D, Duan Y, Chen J, Sun X (2014). Schistosoma japonicum soluble egg antigens facilitate hepatic stellate cell apoptosis by downregulating Akt expression and upregulating p53 and DR5 expression. PLoS Negl Trop Dis.

[6] Moreno M, Gonzalo T, Kok RJ, Sancho-Bru P, van Beuge M, Swart J (2010). Reduction of advanced liver fibrosis by short-term targeted delivery of an angiotensin receptor blocker to hepatic stellate cells in rats. Hepatology.

[7] Wang J, Chu ES, Chen HY, Man K, Go MY, Huang XR (2015). microRNA-29b prevents liver fibrosis by attenuating hepatic stellate cell activation and inducing apoptosis through targeting PI3K/AKT pathway. Oncotarget.

[8] Mishra R, Karande AA (2014). Endoplasmic reticulum stress-mediated activation of p38 MAPK, Caspase-2 and Caspase-8 leads to abrin-induced apoptosis. PLoS One.

[9] Peng Y, Yang H, Zhu T, Tao L (2013). The antihepatic fibrotic effects of fluorofenidone via MAPK signalling pathways. Eur J Clin Investig.

[10] Hernandez-Gea V, Hilscher M, Rozenfeld R, Lim MP, Nieto N, Werner S (2013). Endoplasmic reticulum stress induces fibrogenic activity in hepatic stellate cells through autophagy. J Hepatol.

[11] Huang Y, Li X, Wang Y, Huang C, Li J (2014). Endoplasmic reticulum stress-induced hepatic stellate cell apoptosis through calcium-mediated JNK/P38 MAPK and Calpain/Caspase-12 pathways. Mol Cell Biochem.

[12] Lim MP, Devi LA, Rozenfeld R (2011). Cannabidiol causes activated hepatic stellate cell death through a mechanism of endoplasmic reticulum stress-induced apoptosis. Cell Death Dis.

[13] Fujibayashi T, Hashimoto N, Jijiwa M, Hasegawa Y, Kojima T, Ishiguroet N (2009). Protective effect of geranylgeranylacetone, an inducer of heat shock protein 70, against drug-induced lung injury/fibrosis in an animal model. BMC Pulm Med.

[14] Ikeyama S, Kusumoto K, Miyake H (2001). A non-toxic heat shock protein 70 inducer, geranylgeranylacetone, suppresses apoptosis of cultured rat hepatocytes caused by hydrogen peroxide and ethanol. J Hepatol.

[15] Mao H, Li Z, Zhou Y, Rokutan K, Tashiro S (2008). HSP72 attenuates renal tubular cell apoptosis and interstitial fibrosis in obstructive nephropathy. Am J Physiol Renal Physiol.

[16] He W, Zhuang Y, Wang L, Qi L, Chen B, Wang M (2015). Geranylgeranylacetone attenuates hepatic fibrosis by increasing the expression of heat shock protein 70. Mol Med Rep.

[17] Endo S, Hiramatsu N, Hayakawa K, Okamura M, Kasai A, Tagawa Y (2007). Geranylgeranylacetone, an inducer of the 70-kDa heat shock protein (HSP70), elicits unfolded protein response and coordinates cellular fate independently of HSP70. Mol Pharmacol.

[18] Kluwe J, Pradere JP, Gwak GY, Mencin A, De Minicis S, Osterreicher CH (2010). Modulation of hepatic fibrosis by c-Jun-N-terminal kinase inhibition. Gastroenterology.

[19] Furukawa F, Matsuzaki K, Mori S, Tahashi Y, Yoshida K, Sugano Y (2003). p38 MAPK mediates fibrogenic signal through Smad3 phosphorylation in rat myofibroblasts. Hepatology.

[20] Taimr P, Higuchi H, Kocova E, Rippe RA, Friedman S, Gores GJ (2003). Activated stellate cells express the TRAIL receptor-2/death receptor-5 and undergo TRAIL-mediated apoptosis. Hepatology.

[21] Yu DK, Zhang CX, Zhao SS, Zhang SH, Zhang H, Cai SY (2015). The anti-fibrotic effects of epigallocatechin-3-gallate in bile duct-ligated cholestatic rats and human hepatic stellate LX-2 cells are mediated by the PI3K/Akt/Smad pathway. Acta Pharmacol Sin.

[22] Xu L, Hui AY, Albanis E, Arthur MJ, O'Byrne SM, Blaner WS (2005). Human hepatic stellate cell lines, LX-1 and LX-2:new tools for analysis of hepatic fibrosis. Gut.

[23] Friedman SL (2008). Hepatic stellate cells: protean, multifunctional, and enigmatic cells of the liver. Physiol Rev.

[24] She H, Xiong S, Hazra S, Tsukamoto H (2005). Adipogenic transcriptional regulation of hepatic stellate cells. J Biol Chem.

[25] Hirakawa T, Rokutan K, Nikawa T, Kishi K (1996). Geranylgeranylacetone induces heat shock proteins in cultured guinea pig gastric mucosal cells and rat gastric mucosa. Gastroenterology.

[26] Hoshino T, Suzuki K, Matsushima T, Yamakawa N, Suzuki T, Mizushima T (2013). Suppression of Alzheimer's disease-related phenotypes by geranylgeranylacetone in mice. PLoS One.

[27] Ikeyama S, Kusumoto K, Miyake H, Rokutan K, Tashiro S (2001). Heat shock proteins and mitogen-activated protein kinases in steatotic livers undergoing ischemia-reperfusion: some answers. J Hepatol.

[28] Cazanave SC, Mott JL, Bronk SF, Werneburg NW, Fingas CD, Meng XW, Finnberg N, El-Deiry WS, Kaufmann SH, Gores GJ (2011). Death receptor 5 signaling promotes hepatocyte lipoapoptosis. J Biol Chem.

