# Supplementary figures and images for: Geranylgeranylacetone attenuates fibrogenic activity and induces apoptosis in cultured human hepatic stellate cells and reduces liver fibrosis in carbon tetrachloride-treated mice
Source: BMC Gastroenterol. 2018 Feb 27;18:34. doi: 10.1186/s12876-018-0761-7 (PMC5830074; doi:10.1186/s12876-018-0761-7)

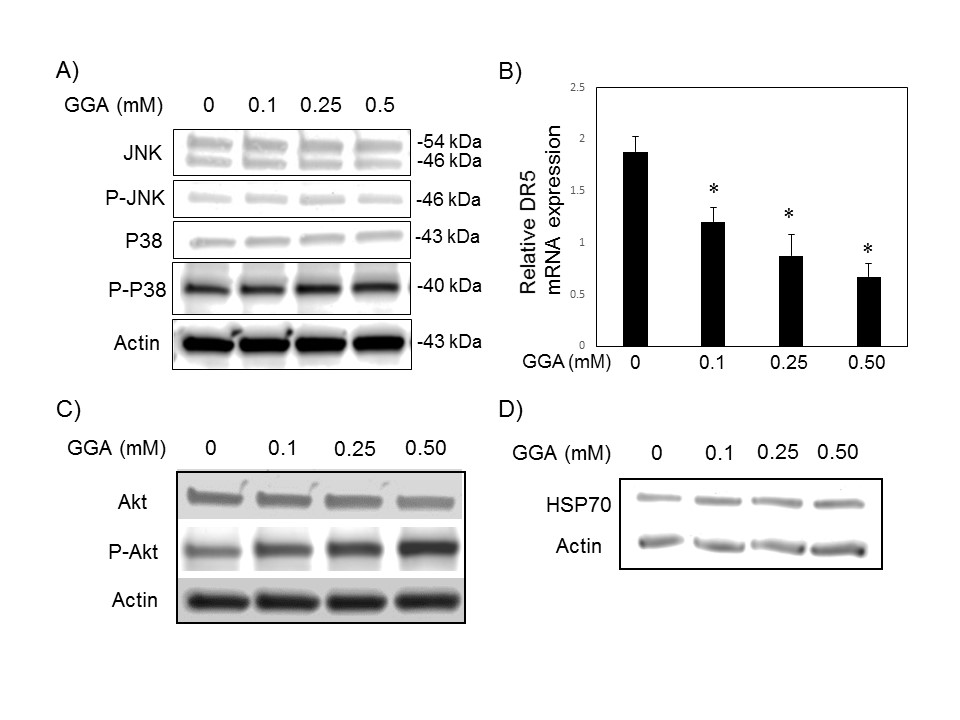

Supplement: Supplementary file 1 — Regarding the activation of Janus N-terminal kinase (JNK) and p38-mitogen activated protein kinase (MAPK), no significant changes were observed in LX2 cells treated with GGA using western blotting analysis (A). GGA suppressed death receptor 5 (DR5) expression in LX2 cells. The expression of DR5 was assessed by real-time PCR. (B) The upregulation of phosphorylated Akt in LX2 cells treated with GGA was observed using western blotting analysis (C). There was no significant change in the expression of HSP70 in LX2 cells treated with GGA and control cells using western blotting analysis (D). In all experiments, LX2 cells were incubated in the presence or absence of GGA (0.10–0.50 mM) for 24 h. * = P < 0.05. (JPEG 73 kb) [file 12876_2018_761_MOESM1_ESM.jpg]
